# Incidence, characteristics, and survival in early- and late-onset colorectal cancer

**DOI:** 10.2340/1651-226X.2025.43349

**Published:** 2025-07-30

**Authors:** Tanja M. Hukkinen, Karri J. Seppä, Nea Malila, Anna H. Lepistö, Camilla C. Böckelman, Laura Koskenvuo

**Affiliations:** a Department of Gastroenterological Surgery, Helsinki University Hospital and University of Helsinki, Helsinki, Finland; b Finnish Cancer Registry, Helsinki, Finland

**Keywords:** colorectal neoplasms, colorectal cancer, survival, incidence, Finland

## Abstract

**Background and purpose:**

To study incidence changes, tumor characteristics, and relative survival (RS) among patients with early- (18–49 years) and late-onset (≥50 years) colorectal cancer (CRC).

**Patient/material and methods:**

In this retrospective registry study, all patients diagnosed with CRC in Finland between 1991 and 2015 were included and followed until death or the end of 2022. Data were extracted from the Finnish Cancer Registry. Changes in incidence as an average annual percentage change as well as age- and sex-standardized RS for CRC were estimated for 5-year periods between 1991–1995 and 2011–2015.

**Results:**

The annual increase in incidence was higher for early-onset CRC versus late-onset CRC (1.2% vs. 0.44%), primarily due to an increase in left-sided colon cancer (2.0%) and rectal cancer (1.5%). Among 59,631 CRC patients, 3,988 (6.7%) had early-onset CRC, of whom 2,073 (52%) were female. Among 55,643 late-onset CRC patients, 27,796 (50%) were female. Among early-onset CRCs, 44% were right-sided, 19% left-sided, and 34% rectal compared with late-onset CRCs, of which 33% were right-sided, 23% left-sided, and 38% rectal. The 5-year RS for early-onset male patients improved from 64% to 72% and for female patients from 69% to 77%, whereas in late-onset patients, survival improved from 51% to 64% among males and from 52% to 67% among females.

**Interpretation:**

The incidence of early-onset CRC is increasing more rapidly than for late-onset CRC. Overall, 5-year RS has improved and is higher in early-onset CRC patients compared with late-onset CRC.

## Introduction

An increase in the number of patients with early-onset colorectal cancer (CRC), defined as diagnosis before the age of 50, has been observed internationally [1]. In 2022, among all new cancer diagnoses (*n* = 37,268) in Finland, 10.9% (*n* = 4,073) were CRC cases, of which 4.9% (*n* = 201) consisted of early-onset cases [2].

Many countries have introduced or are piloting national screening programs for CRC, aiming primarily to decrease CRC-related mortality and ideally the incidence of CRC [3]. Internationally, screening for CRC generally begins at age 50, and, in Finland, the median age of CRC diagnosis is 71.9 years [4]. A decrease in incidence as well as less extensive disease in some countries have already been observed as initial outcomes of screening [5, 6]. Finland’s publicly funded CRC screening program using fecal immunochemical tests (FITs) began in 2022 for individuals aged 60–68, which will expand to include individuals aged 56–74 by 2031 [7]. A FIT result is considered positive when the hemoglobin concentration in the sample is 25 µg Hb/g [8]. Individuals with a positive FIT result are referred for a colonoscopy, arranged by the municipality according to their protocols. Despite screening focusing on older populations, among whom CRC incidence is higher, approximately 10% of CRC cases occur in individuals under the age of 50 [9].

According to a previous report, early-onset CRC is more likely to present at an advanced stage of disease (stage III and IV) compared with late-onset CRC [10]. Despite this, overall survival (OS) among young patients has remained comparable to that among older age groups [11]. Moreover, estimates of reported trends of a rising incidence among early-onset CRC in many countries indicate that, within the next 10 years in the US, 10–12% of patients with colon cancer and 25% of patients with rectal cancer will be diagnosed in patients younger than 50 [9, 12, 13]. In Finland, figures suggest that, by 2040, the relative incidence of early-onset CRC among females will increase by 28%, and decrease among males by 3% [14]. To our knowledge, more extensive research on changes in incidence among histological types and CRC locations has not been previously reported. In this nationwide registry study, we aimed to investigate trends in the incidence of early-onset CRC, the trend in 5-year relative survival (RS) according to histology, stage, and location of the tumor in Finland, and to compare those outcomes with those among late-onset CRC patients.

## Patients and methods

### Study cohort

In this study, we included all incident CRCs (ICD-10 C18.0–20.9) diagnosed in Finland from 1991–2015 aged 18 or older, following patients until death or through the end of 2022. We received our data from the Finnish Cancer Registry (FCR) and included patient gender, age at diagnosis, tumor-specific data (histology, stage, and location), and the time of death. FCR is a comprehensive, nationwide cancer registry, which collects data from all healthcare providers in Finland. Finland has a universal and tax-funded healthcare system, which provides access to medical services to all residents. Cancer cases were linked to the Population Information System, which includes data on individuals’ dates of birth and death. Statistics Finland provided data on the background population and mortality rates in Finland (stratified by both sex and 1-year groups by age and calendar year). Here, we classified cancer as early-onset if diagnosed at 18–49 years of age and late-onset if diagnosed at 50 years or older, and excluded patients under the age of 18 (Supplementary Figure 1).

### Clinical characteristics

We classified tumors as right-sided if located between the cecum and the end of the transverse colon. After 2007, appendiceal cancer was more easily distinguished from right-sided colon cancer in the FCR data, which we separated from right-sided colon cancer in the 5-year survival analyses. Previously, appendiceal cancer was included in the right-sided colon cancers. We considered tumors left-sided if located from the splenic flexure to the end of the sigmoid colon, with tumors in the rectosigmoid junction included in the rectum subgroup. Finally, we classified patients with an unknown tumor location as ‘others’.

We divided our data into histological groups of adenocarcinoma and neuroendocrine tumors (NET) according to the International Classification of Diseases (ICD) for oncology morphology [15], grouping together the remaining cases as ‘others’. In classifying the tumor stage, FCR uses its own classification system, designed to categorize all cancer cases as accurately as possible, even when data are partially incomplete [16]. Localized cancer indicates no spread to nearby lymph nodes or beyond (TNM [tumor, node, metastasis] stages I–II [17]), while nonlocalized disease indicates spread to nearby lymph nodes or more distant regions (TNM stages III–IV).

### Statistics

Our primary comparison of interest in this study focused on early-onset patients versus late-onset patients. We calculated age-standardized incidence rates for CRC in 5-year calendar periods (from 1991–1995 to 2011–2015) based on 5-year age groups adjusted to the World Standard Population [18]. We estimated the average annual percentage change (AAPC) using a Poisson regression model, modelling the logarithm of the incidence rate as a function of the age group and calendar year. We assessed the difference in AAPC between early- and late-onset cancers using the likelihood ratio test, comparing the model with age group-specific trend parameters to the model with a single-trend parameter. In addition, we used joinpoint regression to estimate piecewise trends (annual percentage change, APC) in incidence adjusted for age and sex, reporting only statistically significant changes in incidence based on the Davies test. We estimated age- and sex-standardized RS for the same 5-year periods using the age distribution of patients diagnosed during the entire 1991–2015 time period as weights (using six age groups: 18–39, 40–49, 50–59, 60–69, 70–79, and 80 and older), and employing the Ederer II method [19]. We also used the Poisson regression model for RS to compare survival between early- and late-onset patients based on the likelihood ratio test [20]. Survival models included six intervals of follow-up time (< 4 months, 4 to < 12 months, and four annual intervals from 1 to 5 years, respectively). To compare patients diagnosed in 2011–2015, we used a model with an additional parameter for the effect of age at onset to compare that to one without the parameter. In the comparison of the trend over the 5-year periods from 1991–1995 to 2011–2015, we added age at diagnosis (the same groups as above), their interactions with the follow-up time intervals, and a numerical variable for the effect of the five periods in the models, and compared the model with its own trend parameter for the effect of age at onset to a model with a common trend parameter. To compare patient characteristics between early- and late-onset CRC cases, we estimated the differences in proportions with 95% confidence intervals (CIs). For all statistical analyses, we relied on the statistical program R, version 4.0.5 using the popEpi package (version 0.4.10) and the segmented package (version 1.3.3).

### Permissions

The National Institute of Health and Welfare approved the study protocol (THL/722/5.05.00/2018, extension THL/3562/14.06.00/2022).

## Results

### Trends in incidence

**Figure 1:**
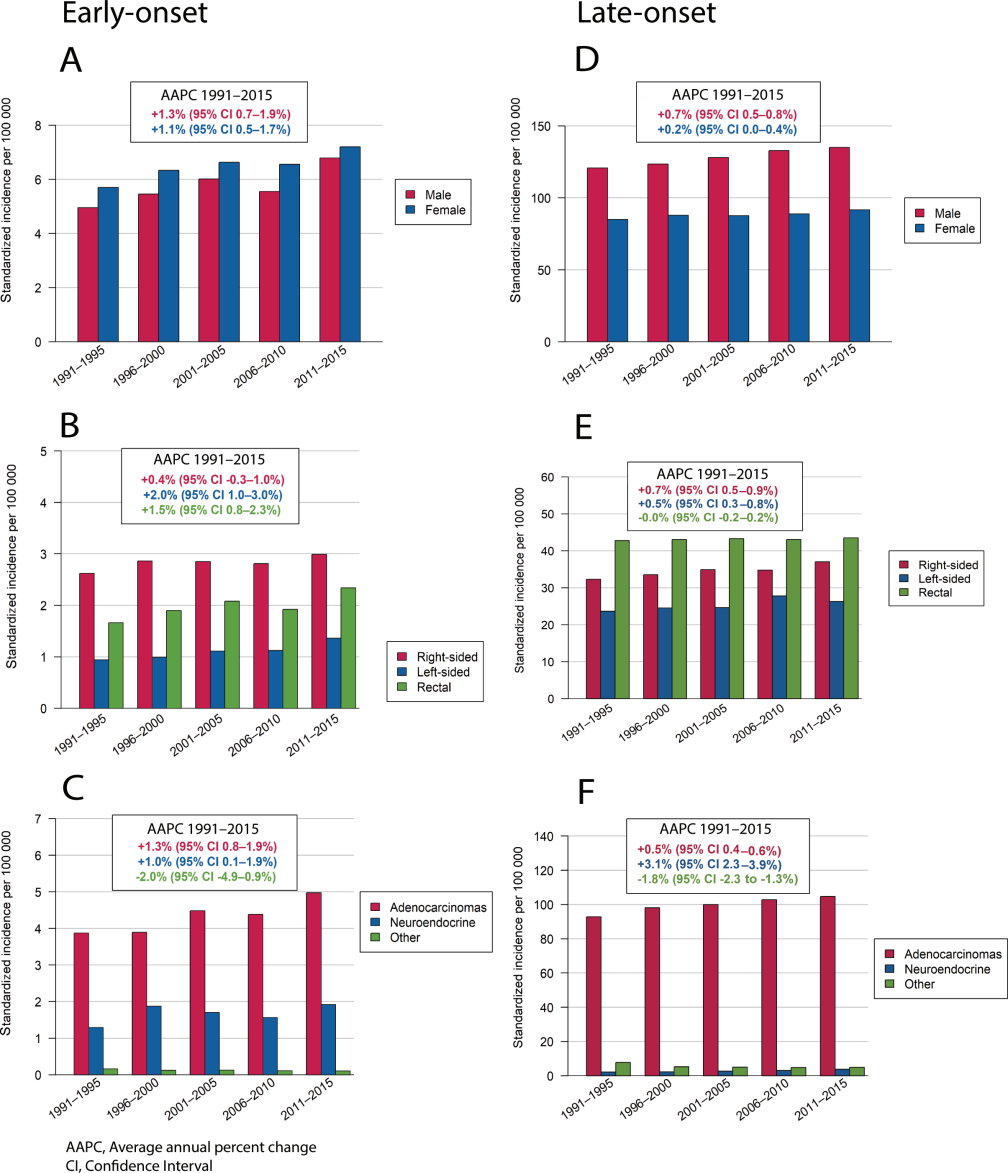
Incidence for patients with early-onset colorectal cancer during 1991–2015 according to (A) sex, (B) tumor location, and (C) histology and late-onset colorectal cancer according to (D) sex, (E) tumor location, and (F) histology.

The incidence of early-onset CRC increased more than late-onset CRC (AAPC 1.19% vs. 0.44%, difference 0.75%; Table 1). We also observed a steady increase in the incidence of early-onset CRC annually among males (AAPC 1.3% [95% CI 0.67–1.94]) and among females (AAPC 1.08% [95% CI 0.48–1.69]) during the study period (Figure 1, Supplemental Table 1). By contrast, the increase in incidence for late-onset CRC for males (AAPC 0.65% [95% CI 0.48–0.82]) was higher than that among females (AAPC 0.21% [95% CI 0.04–0.37]). The most notable annual increase in incidence among early-onset CRC was observed in adenocarcinomas (AAPC 1.34% [95% CI 0.83–1.85]), whereas it was most notable in NETs in late-onset CRC (AAPC 3.05% [95% CI 2.25–3.85]). The annual incidence of both early- and late-onset localized CRC decreased (AAPC −2.77% [95% CI −3.49 to −2.05] and AAPC −2.2% [95% CI −2.39 to −2.00]), respectively, while the incidence for nonlocalized CRC increased (AAPC 2.14% [95% CI 1.47–2.81] and AAPC 1.18% [95% CI 1.00–1.35]). The incidence of early-onset right-sided colon cancer remained stable (AAPC 0.35% [95% CI −0.30 to 1.01]), whereas we detected a notable increase for left-sided colon cancer (AAPC 1.96% [95% CI 0.95–2.98]) and for rectal cancer (AAPC 1.52% [95% CI 0.76–2.28]; Figure 1, Table 1).

**Table 1:** Average annual percent change (AAPC) for early- and late-onset colorectal cancer incidence rate from 1991 to 2015 and difference between early- and late-onset incidence.

	18–49		≥ 50 years			
	AAPC	95% CI	AAPC	95% CI	Difference	95% CI
**All**	1.19	(0.75 to 1.63)	0.44	(0.32 to 0.56)	0.75	(0.30 to 1.20)
**Sex**						
Male	1.30	(0.67 to 1.94)	0.65	(0.48 to 0.82)	0.65	(0.00 to 1.31)
Female	1.08	(0.48 to 1.69)	0.21	(0.04 to 0.37)	0.88	(0.25 to 1.51)
**Tumor location**						
Right-sided colon	0.35	(−0.30 to 1.01)	0.67	(0.47 to 0.87)	−0.32	(−1.00 to 0.37)
Left-sided colon	1.96	(0.95 to 2.98)	0.53	(0.29 to 0.78)	1.43	(0.38 to 2.47)
Rectum	1.52	(0.76 to 2.28)	−0.03	(−0.22 to 0.15)	1.55	(0.77 to 2.33)
Other	4.68	(2.19 to 7.23)	2.15	(1.62 to 2.69)	2.53	(−0.05 to 5.11)
**Histology**						
Adenocarcinoma	1.34	(0.83 to 1.85)	0.52	(0.39 to 0.64)	0.82	(0.30 to 1.35)
Neuroendocrine tumor	1.02	(0.14 to 1.91)	3.05	(2.25 to 3.85)	−2.03	(−3.22 to −0.83)
Other	−2.04	(−4.92 to 0.93)	−1.78	(−2.25 to −1.31)	−0.26	(−3.23 to 2.70)
**Stage**						
Localized	−2.77	(−3.49 to −2.05)	−2.20	(−2.39 to −2.00)	−0.57	(−1.32 to 0.17)
Nonlocalized	2.14	(1.47 to 2.81)	1.18	(1.00 to 1.35)	0.96	(0.27 to 1.66)
Unknown	6.18	(5.14 to 7.22)	3.25	(2.98 to 3.52)	2.93	(1.85 to 4.00)

CI: confidence interval.

### Patient characteristics

Among all patients (*n* = 59,631), we identified 3,988 patients with early-onset CRC and 55,643 patients with late-onset CRC (Table 2). Among early-onset patients, 2,073 (52.0%) were female, and among late-onset patients 27,796 (50.0%) were female. Early-onset patients were less likely to have adenocarcinoma based on the tumor histology compared with late-onset patients (*n* = 2,941 [73.7%] vs. *n* = 51,067 [91.8%]), whereas a neuroendocrine histology appeared more common among early-onset patients (*n* = 961 [24.1%] vs. *n* = 1,328 [2.4%], difference 21.7%). A right-sided tumor location among early-onset patients was more common compared with late-onset patients (*n* = 1,750 [43.9%] vs. *n* = 18,587 [33.4%]), while left-sided and rectal tumors less frequently occurred among early-onset patients (*n* = 757 [19.0%] vs. *n* = 12,897 [23.2%], difference −4.2%, and *n* = 1,347 [33.8%] vs. *n* = 21,329 [38.3%], difference −4.6%; Table 2).

**Table 2:** Patient characteristics for early- and late-onset colorectal cancer patients diagnosed in Finland, 1991–2015.

Patient characteristic			
	18–49	≥ 50	Difference in proportion, % (95% CI)
	*n* (%)	*n* (%)	
**All**	3,988 (100.0)	55,643 (100.0)	
**Gender**			
Males	1,915 (48.0)	27,847 (50.0)	−2.0 (−3.6 to −0.4)
Females	2,073 (52.0)	27,796 (50.0)	2.0 (0.4 to 3.6)
**Stage**			
Localized	1,406 (35.2)	18,304 (32.9)	2.4 (0.8 to 3.9)
Nonlocalized	1,741 (43.7)	25,924 (46.6)	−2.9 (−4.5 to −1.3)
Unknown	841 (21.1)	11,415 (20.5)	0.6 (−0.7 to 1.9)
**Tumor location**			
Right-sided colon	1,750 (43.9)	18,587 (33.4)	10.5 (8.9 to 12.1)
Right-sided colon, excluding appendix	1,345 (33.7)	17,828 (32.0)	1.7 (0.2 to 3.2)
Appendix	405 (10.2)	759 (1.4)	8.8 (7.8 to 9.7)
Left-sided colon	757 (19.0)	12,897 (23.2)	−4.2 (−5.5 to −2.9)
Rectum	1,347 (33.8)	21,329 (38.3)	−4.6 (−6.1 to −3.0)
Others	134 (3.3)	2,830 (5.1)	−1.7 (−2.3 to −1.1)
**Histology**			
Adenocarcinoma	2,941 (73.7)	51,067 (91.8)	−18.0 (−19.4 to −16.6)
Neuroendocrine tumor	961 (24.1)	1,328 (2.4)	21.7 (20.4 to 23.0)
Other	86 (2.2)	3,248 (5.8)	−3.7 (−4.2 to −3.2)

CI: confidence interval.

### 5-year relative survival

**Figure 2:**
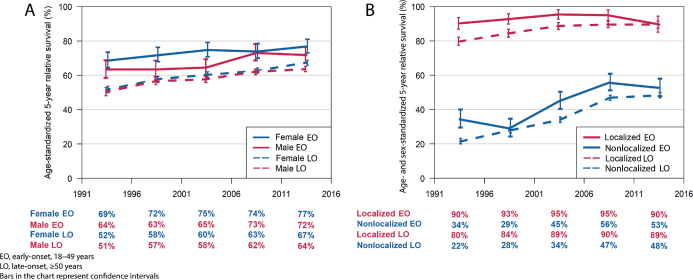
Five-year relative survival for early- and late-onset colorectal cancer patients diagnosed during 1991–2015 according to (A) sex and (B) stage.

For female patients, the 5-year RS for early-onset CRC in 2011–2015 was 76.9% (95% CI 73.1–80.9) versus 67.4% (95% CI 66.0–68.9) for late-onset patients. For male patients, the 5-year RS for early-onset CRC in 2011–2015 was 71.7% (95% CI 67.5–76.3) versus 63.7% (95% CI 62.1–65.2) for late-onset patients. We found that the 5-year RS among early-onset CRC patients with localized disease has remained stable, varying from 90.1% (95% CI 86.7–93.6) and 89.6% (95% CI 85.0–94.4), while in late-onset patients, RS increased from 79.5% (95% CI 77.1–82.0) to 89.4% (95% CI 87.4–91.6) during the study period. For early-onset patients with nonlocalized disease, RS increased from 34.1% (95% CI 29.0–40.0) to 53.0% (95% CI 48.1–58.4), and for late-onset patients from 21.6% (95% CI 20.0–23.4) to 48.4% (95% CI 46.9–50.0; Figure 2, Table 3, and Supplemental Table 2).

**Table 3:** Age- and sex-standardized 5-year relative survival (%) for early- and late-onset colorectal cancer patients diagnosed between 2011 and 2015 (95% CI).

	18–49 years	≥ 50 years	Difference
**Sex**			
Male	71.7 (67.5 to 76.3)	63.7 (62.1 to 65.2)	8.1 (3.4 to 12.8)
Female	76.9 (73.1 to 80.9)	67.4 (66.0 to 68.9)	9.5 (5.3 to 13.7)
**Stage**			
Localized	89.6 (85.0 to 94.4)	89.4 (87.4 to 91.6)	0.1 (−5.0 to 5.3)
Nonlocalized	53.0 (48.1 to 58.4)	48.4 (46.9 to 50.0)	4.6 (−0.8 to 9.9)
Unknown	90.9 (87.5 to 94.3)	73.7 (72.0 to 75.5)	17.1 (13.3 to 21.0)
**Tumor location**			
Right-sided colon	77.1 (72.4 to 82.2)	66.3 (64.5 to 68.2)	10.8 (5.6 to 16.1)
Right-sided colon excluding appendix	63.2 (55.4 to 72.1)	65.5 (63.6 to 67.5)	−2.3 (−10.9 to 6.2)
Left-sided colon	67.0 (59.8 to 75.1)	64.2 (62.0 to 66.5)	2.8 (−5.1 to 10.8)
Rectum	73.6 (68.7 to 78.9)	65.4 (63.7 to 67.2)	8.2 (2.8 to 13.5)
Other	62.3 (46.8 to 83.0)	62.2 (58.1 to 66.6)	0.1 (−18.2 to 18.5)
**Histology**			
Adenocarcinoma	69.4 (65.7 to 73.2)	67.9 (66.8 to 69.0)	1.5 (−2.4 to 5.4)
Neuroendocrine tumor	86.5 (80.1 to 93.4)	62.8 (55.2 to 71.6)	23.6 (13.1 to 34.2)
Other	70.9 (51.3 to 98.0)	19.0 (15.1 to 24.0)	51.8 (28.5 to 75.2)

CI: confidence interval.

For 2011–2015, the RS for early-onset patients with right-sided colon cancer including appendiceal cancer was 77.1% (95% CI 72.4–82.2). However, when we excluded appendiceal cancers, RS fell to 63.2% (95% CI 55.4–72.1). In late-onset patients, the RS for right-sided colon cancer was 66.3% (95% CI 64.5–68.2) including appendiceal tumors, and 65.5% (95% CI 63.6–67.5) excluding appendiceal tumors. In early-onset patients, the RS for left-sided colon cancer patients increased from 50.8% (95% CI 42.8–60.1%) to 67.0% (95% CI 59.8–75.1%), and in rectal cancer patients from 63.2% (95% CI 57.4–69.7%) to 73.6% (95% CI 68.7–78.9%). Among late-onset patients, RS increased from 52.3% (95% CI 49.2–55.6) to 64.2% (95% CI 62.0–66.5) for left-sided colon cancer, and from 49.6% (95% CI 47.3–51.9) to 65.4% (95% CI 63.7–67.2) for rectal cancer.

## Discussion and conclusion

This nationwide study revealed an increase in incidence among early-onset CRC in Finland from 1991 to 2015, consistent with earlier studies [1, 21]. The most substantial subgroup increase for early-onset patients was observed in adenocarcinomas, as well as in left-sided and rectal tumors. In addition, we observed a large increase in incidence among early-onset CRC patients with nonlocalized disease. While late-onset CRC showed an overall increase in incidence, it was less pronounced than that for early-onset CRC. Early-onset female patients exhibited a higher RS than late-onset female patients, as did early-onset male patients compared with late-onset male patients. We also observed a similar RS among patients with a localized tumor in both early- and late-onset CRC, an important finding given the aging population [22]. The most common tumor location was the right-sided colon for early-onset patients and the rectum for late-onset patients.

We found that the annual average increase in incidence for early-onset CRC aligned with other large cohort studies reporting annual increases of 0.70–3.97% [1, 23, 24]. This was coupled with an increase in nonlocalized disease (stages III–IV), while the incidence of localized disease (stages I–II) decreased. A previous report also observed an increase in disease incidence in early-onset stage III and IV, while the incidence for stages I and II has decreased over time [23]. This trend may reflect a ‘stage migration’, whereby improved staging techniques result in previously localized tumors being reclassified as nonlocalized [25]. We also detected a substantial increase in late-onset CRC incidence, albeit significantly smaller than that for early-onset CRC. Previous studies similarly highlighted a higher increase in incidence for early-onset CRC compared with late-onset CRC [1, 26]. Moreover, we found an increasing incidence in late-onset colon cancer, while rectal cancer incidence remained more stable. By contrast, an increase in incidence for early-onset CRC was previously reported for distal colon and rectal cancer, which we also found in our study [27]. Furthermore, in some countries, the annual incidence of late-onset CRC has begun declining, purportedly related to colorectal screening programs [9]. The impact of screening in Finland, which began in 2022, is expected to emerge in the coming years. Since screening for CRC in Finland had not yet been introduced when the data for this study were collected, the effect of screening remains unclear. To our knowledge, no previous nationwide study focusing on early-onset CRC has been conducted in Finland. Earlier studies, however, focused on all age groups [4, 28]. We argue that this study can serve as a benchmark for evaluating the effects of CRC screening once relevant outcome data become available.

The incidence of early-onset rectal NETs has reportedly increased significantly, while colon NETs appear less common [27]. A study examining more than 500,000 CRC patients over the age of 20 found that the rapid rise in early-onset cases was primarily due to the increase in NETs (AAPC 2.65%), with adenocarcinomas also increasing (AAPC 0.91%) [29]. This differs from our findings, whereby the most notable AAPC in early-onset CRC was observed in adenocarcinomas, while NETs showed a more substantial increase in late-onset CRCs.

As the incidence of early-onset CRCs increases, early detection and treatment become increasingly crucial. A recent study found that pre-diagnostic symptoms, such as rectal bleeding and abdominal pain, appeared 3 months to 2 years prior to diagnosis in one-fifth of patients, while symptoms appeared within 3 months of diagnosis in half of patients [30]. Younger patients experience longer delays in diagnosis and are more often diagnosed at a more advanced stage of disease [10, 31]. Such findings differ from our results, whereby we found that both early- and late-onset CRC cases were predominantly nonlocalized compared with localized cases. This highlights the importance of clinicians considering the possibility of CRC in patients under 50. The rising incidence trends for early-onset CRC may lead to many psychosocial challenges, underpinning the need for interventions such as fertility preservation and family planning for young patients [32].

Earlier studies have reported varying findings regarding the prognosis of early- versus late-onset patients [33]. The 5-year RS for early-onset patients in our study was consistent with previously reported rates of 78–94% for localized disease [34]. A large cohort study found that early-onset patients, despite more aggressive treatment, experienced minimal survival benefit compared with late-onset patients, who underwent less intensive treatment [35]. Another study reported a higher disease-specific mortality among early-onset stage III patients, whereas the 5-year OS in that study was similar for early- and late-onset patients [36]. In our study, early-onset patients with nonlocalized disease exhibited a better RS than late-onset patients. An earlier report detected improvements among early-onset patients in RS for both nonlocalized and localized disease [23]. Another study indicated a greater increase in survival for localized disease, notably not differentiating between early- and late-onset cases, potentially impacting the results [37]. By contrast, our findings revealed stable survival rates throughout the follow-up period for localized disease.

Improvements in RS were observed across all CRC locations for both early- and late-onset patients. The RS for right-sided colon cancer was influenced by appendiceal tumors among early-onset patients until 2007, after which appendiceal tumors were excluded from the right-sided colon cancer group. After the exclusion of appendiceal cancer, the RS rates dropped for early-onset right-sided colon cancer. We did not observe the same trend for late-onset patients, possibly due to their small proportion in this patient group. Previously, a study among 1,652 early-onset appendiceal cancer patients found a survival rate of 63–76% depending on race or ethnicity [38]. The number of patients with early-onset appendiceal cancer in our data was significant, potentially influencing the results when included.

The strength of this study is its nationwide registry-based approach, which provided comprehensive data on CRC with a high completeness rate of 97.4% [39]. The registry receives histological reports from all pathology laboratories in Finland, ensuring a thorough classification of CRC and a reliable identification of tumor subsites within the colorectum. Since survival for CRC patients is good, the long follow-up period in our study represents a strength, contributing to a more accurate assessment of long-term survival and is of importance for understanding patient outcomes. However, this study’s registry-based nature means we also lack detailed information on clinical characteristics, patient treatments, and comorbidities. Despite this limitation, the large cohort and long follow-up period offer valuable insights into CRC incidence and outcomes on a national level in a Nordic country.

In conclusion, the incidence of early-onset CRC is rising. However, 5-year RS has improved for both early- and late-onset CRC patients over the study period. Early-onset CRC is often located in the right-side of the colon with adenocarcinoma representing the most common tumor type, although the incidence of NETs is also increasing. We conclude that late-onset CRC patients present more often with tumors in the rectum, and their survival rates are generally comparable to those among early-onset patients.

## Supplementary Material



## Data Availability

All patients included in this study population were identified by the authorities at FCR and the data were anonymized. Individual patients will neither benefit nor be harmed by inclusion in this study. All physicians involved in the study followed the principles of professional patient confidentiality. Ethical review and patient informed consent were thus waived due to the retrospective registry nature of the study and the large study population.
