# Supplementary material for: Incidence, characteristics, and survival in early- and late-onset colorectal cancer
Source: Acta Oncol. 2025 Jul 30;64:43349. doi: 10.2340/1651-226X.2025.43349 (PMC12320144; doi:10.2340/1651-226X.2025.43349)

Supplementary material has been published as submitted. It has not been copyedited, or typeset by Acta Oncologica

STROBE Statement—Checklist of items that should be included in reports of ***cohort studies***

	Item No	Recommendation	Page No
**Title and abstract**	1	(*a*) Indicate the study's design with a commonly used term in the title or the abstract	1,3
		(*b*) Provide in the abstract an informative and balanced summary of what was done and what was found	3
**Introduction**			
Background/rationale	2	Explain the scientific background and rationale for the investigation being reported	4
Objectives	3	State specific objectives, including any prespecified hypotheses	4
**Methods**			
Study design	4	Present key elements of study design early in the paper	4,5
Setting	5	Describe the setting, locations, and relevant dates, including periods of recruitment, exposure, follow-up, and data collection	4,5
Participants	6	(*a*) Give the eligibility criteria, and the sources and methods of selection of participants. Describe methods of follow-up	4,5
		(*b*) For matched studies, give matching criteria and number of exposed and unexposed	
Variables	7	Clearly define all outcomes, exposures, predictors, potential confounders, and effect modifiers. Give diagnostic criteria, if applicable	4,5
Data sources/ measurement	8*	For each variable of interest, give sources of data and details of methods of assessment (measurement). Describe comparability of assessment methods if there is more than one group	4,5
Bias	9	Describe any efforts to address potential sources of bias	4,5,9
Study size	10	Explain how the study size was arrived at	4,5
Quantitative variables	11	Explain how quantitative variables were handled in the analyses. If applicable, describe which groupings were chosen and why	4,5
Statistical methods	12	(*a*) Describe all statistical methods, including those used to control for confounding	4,5,6
		(*b*) Describe any methods used to examine subgroups and interactions	
		(*c*) Explain how missing data were addressed	
		(*d*) If applicable, explain how loss to follow-up was addressed	
		(*e*) Describe any sensitivity analyses	
**Results**			
Participants	13*	(a) Report numbers of individuals at each stage of study—eg numbers potentially eligible, examined for eligibility, confirmed eligible, included in the study, completing follow-up, and analysed	6,7
		(b) Give reasons for non-participation at each stage	
		(c) Consider use of a flow diagram	
Descriptive data	14*	(a) Give characteristics of study participants (eg demographic, clinical, social) and information on exposures and potential confounders	6,7
		(b) Indicate number of participants with missing data for each variable of interest	
		(c) Summarise follow-up time (eg, average and total amount)	
Outcome data	15*	Report numbers of outcome events or summary measures over time	6,7

Main results	16	(*a*) Give unadjusted estimates and, if applicable, confounder-adjusted estimates and their precision (eg, 95% confidence interval). Make clear which confounders were adjusted for and why they were included<br>(*b*) Report category boundaries when continuous variables were categorized<br>(*c*) If relevant, consider translating estimates of relative risk into absolute risk for a meaningful time period	6,7
Other analyses	17	Report other analyses done—eg analyses of subgroups and interactions, and sensitivity analyses	6,7
**Discussion**			
Key results	18	Summarise key results with reference to study objectives	7,9
Limitations	19	Discuss limitations of the study, taking into account sources of potential bias or imprecision. Discuss both direction and magnitude of any potential bias	9
Interpretation	20	Give a cautious overall interpretation of results considering objectives, limitations, multiplicity of analyses, results from similar studies, and other relevant evidence	7,8,9
Generalisability	21	Discuss the generalisability (external validity) of the study results	7,8,9
**Other information**			
Funding	22	Give the source of funding and the role of the funders for the present study and, if applicable, for the original study on which the present article is based	9

*Give information separately for exposed and unexposed groups.

Note: An Explanation and Elaboration article discusses each checklist item and gives methodological background and published examples of transparent reporting. The STROBE checklist is best used in conjunction with this article (freely available on the Web sites of PLoS Medicine at http://www.plosmedicine.org/, Annals of Internal Medicine at http://www.annals.org/, and Epidemiology at http://www.epidem.com/). Information on the STROBE Initiative is available at http://www.strobe-statement.org.

Supplemental Table 1. Annual percentage change (APC) of early- and late-onset colorectal cancer incidence in 1991–2015, based on joinpoint regression

	**18–49**			**≥50**		
	**Period**	**APC**	**95% CI[1]**	**Period**	**APC**	**95% CI**
All	1991–2015	1.19	(0.75–1.63)	1991–2015	0.44	(0.32–0.56)
**Sex**						
Male	1991–2015	1.30	(0.67–1.94)	1991–2015	0.65	(0.48–0.82)
Female	1991–2015	1.08	(0.48–1.69)	1991–2015	0.21	(0.04–0.37)
**Tumor location**						
Right-sided colon	1991–2015	0.35	(-0.30– 1.01)	1991–2013	0.43	(0.18–0.68)
	-	-	-	2013–2015	6.84	(2.33–11.55)
Left-sided colon	1991–2015	1.96	(0.95–2.98)	1991–2015	0.53	(0.29–0.78)
Rectum	1991–2015	1.52	(0.76–2.28)	1991–2015	-0.03	(-0.22–0.15)
Other	1991–2015	4.68	(2.19–7.23)	1991–2013	2.96	(2.34–3.58)
	-	-	-	2013–2015	-24.23	(-40.07 to -4.20)
**Histology**						
Adenocarcinoma	1991–2015	1.34	(0.83–1.85)	1991–2015	0.52	(0.39–0.64)
Neuroendocrine tumor	1991–1993	36.18	(4.62–77.27)	1991–2015	3.05	(2.25–3.85)
	1993–2015	0.22	(-0.82–1.27)	-	-	-
Other	1991–2015	-2.04	(-4.92–0.93)	1991–1998	-6.89	(-9.88 to -3.81)
	-	-	-	1998–2015	-0.33	-
**Stage**						
Localized	1991–1996	6.33	(-1.44–14.72)	1991–2009	-0.99	(-1.29 to -0.69)
	1996–2015	-4.18	(-5.19 to -3.15)	2009–2015	-9.30	(-10.94 to -7.64)
Nonlocalized	1991–2015	2.14	(1.47–2.81)	1991–2009	2.41	(2.14–2.69)
	-	-	-	2009–2015	-4.48	(-5.71 to -3.23)
Unknown	1991–2010	1.85	(0.21–3.53)	1991–2009	-1.78	(-2.22 to -1.34)
	2010–2015	26.79	(18.91–35.19)	2009–2015	21.85	(19.80–23.93)

[1]CI, confidence interval

Supplemental Table 2. Age- and sex-standardized 5-year relative survival (%) for early- and late-onset colorectal cancer patients during 1991–2015 (95% CI) [1]

		18–49 years	≥50 years	Difference	*p-value*[2]
**Sex**					
Male					*0.89*
	1991–1995	63.5 (58.5–68.9)	50.5 (48.2–52.9)	13.0 (7.3–18.7)	
	1996–2000	63.3 (58.6–68.5)	56.8 (54.5–59.3)	6.5 (1.0–12.0)	
	2001–2005	64.5 (59.9–69.5)	57.7 (55.7–59.7)	6.9 (1.7–12.1)	
	2006–2010	73.2 (68.7–78.1)	62.1 (60.3–63.8)	11.2 (6.1–16.2)	
	2011–2015	71.7 (67.5–76.3)	63.7 (62.1–65.2)	8.1 (3.4–12.8)	
Female					*0.41*
	1991–1995	68.6 (64.1–73.5)	51.7 (49.9–53.6)	16.9 (11.9–22.0)	
	1996–2000	71.9 (67.8–76.3)	57.6 (55.8–59.4)	14.3 (9.7–18.9)	
	2001–2005	74.8 (70.8–79.0)	60.4 (58.8–62.1)	14.4 (9.9–18.8)	
	2006–2010	73.9 (69.8–78.3)	62.8 (61.3–64.3)	11.1 (6.6–15.7)	
	2011–2015	76.9 (73.1–80.9)	67.4 (66.0–68.9)	9.5 (5.3–13.7)	
**Stage**					
Localized					*0.13*
	1991–1995	90.1 (86.7–93.6)	79.5 (77.1–82.0)	10.6 (6.3–14.8)	
	1996–2000	92.6 (89.6–95.7)	84.9 (82.4–87.4)	7.7 (3.8–11.7)	
	2001–2005	95.3 (92.6–98.2)	88.5 (86.5–90.6)	6.8 (3.3–10.3)	
	2006–2010	94.9 (91.8–98.2)	89.8 (87.9–91.7)	5.2 (1.5–8.9)	
	2011–2015	89.6 (85.0–94.4)	89.4 (87.4–91.6)	0.1 (-5.0–5.3)	
Nonlocalized					*0.55*
	1991–1995	34.1 (29.0–40.0)	21.6 (20.0–23.4)	12.5 (6.7–18.2)	
	1996–2000	29.4 (24.4–35.4)	28.1 (26.3–30.0)	1.3 (-4.4–7.1)	
	2001–2005	45.2 (40.3–50.7)	34.1 (32.5–35.7)	11.2 (5.7–16.6)	
	2006–2010	55.8 (50.9–61.1)	47.2 (45.7–48.7)	8.6 (3.3–13.9)	
	2011–2015	53.0 (48.1–58.4)	48.4 (46.9–50.0)	4.6 (-0.8–9.9)	
Unknown					*0.05*
	1991–1995	78.5 (70.7–87.2)	55.5 (51.8–59.5)	23.0 (13.9–32.1)	
	1996–2000	83.2 (76.7–90.2)	66.8 (63.8–69.9)	16.4 (9.0–23.8)	
	2001–2005	82.7 (76.2–89.7)	63.3 (60.2–66.5)	19.4 (12.0–26.9)	
	2006–2010	89.2 (82.6–96.3)	60.6 (57.3–64.1)	28.6 (21.0–36.2)	
	2011–2015	90.9 (87.5–94.3)	73.7 (72.0–75.5)	17.1 (13.3–21.0)	
**Tumor location**					
Right-sided colon					*0.37*
	1991–1995	73.5 (68.8–78.6)	52.8 (50.1–55.7)	20.7 (15.1–26.3)	
	1996–2000	72.8 (68.0–77.9)	59.9 (57.3–62.6)	12.9 (7.3–18.5)	
	2001–2005	73.0 (68.1–78.3)	59.7 (57.5–62.0)	13.3 (7.8–18.8)	
	2006–2010	79.0 (74.5–83.8)	61.4 (59.4–63.5)	17.6 (12.5–22.6)	
	2011–2015	77.1 (72.4–82.2)	66.3 (64.5–68.2)	10.8 (5.6–16.1)	
Right-sided colon excluding appendix					*0.15*
	2007–2010	69.3 (61.4–78.3)	61.2 (58.9–63.7)	8.1 (-0.6–16.8)	
	2011–2015	63.2 (55.4–72.1)	65.5 (63.6–67.5)	-2.3 (-10.9–6.2)	

Left-sided colon				*0.12*
1991–1995	50.8 (42.8–60.1)	52.3 (49.2–55.6)	-1.6 (-10.8–7.6)	
1996–2000	48.3 (39.8–58.4)	56.2 (53.3–59.4)	-8 (-17.7–1.8)	
2001–2005	67.2 (59.7–75.6)	59.3 (56.6–62.0)	7.9 (-0.5–16.3)	
2006–2010	65.9 (58.4–74.2)	63.2 (60.9–65.6)	2.6 (-5.6–10.9)	
2011–2015	67.0 (59.8–75.1)	64.2 (62.0–66.5)	2.8 (-5.1–10.8)	
Rectum				*0.42*
1991–1995	63.2 (57.4–69.7)	49.6 (47.3–51.9)	13.7 (7.1–20.3)	
1996–2000	67.7 (62.3–73.6)	54.7 (52.5–57.1)	13.0 (6.9–19.1)	
2001–2005	66.3 (60.7–72.3)	58.5 (56.5–60.7)	7.7 (1.6–13.9)	
2006–2010	72.2 (66.6–78.2)	63.6 (61.7–65.6)	8.6 (2.5–14.7)	
2011–2015	73.6 (68.7–78.9)	65.4 (63.7–67.2)	8.2 (2.8–13.5)	
Other				*0.06*
1991–1995	86.8 (75.0–100.0)	44.6 (38.1–52.2)	42.2 (27.8–56.7)	
1996–2000	69.6 (52.2–93.0)	58.7 (52.0–66.2)	11.0 (-10.4–32.3)	
2001–2005	48.5 (35.0–67.1)	50.5 (45.3–56.4)	-2.1 (-18.8–14.7)	
2006–2010	52.8 (38.2–73.1)	50.6 (45.6–56.2)	2.2 (-15.7–20.2)	
2011–2015	62.3 (46.8–83.0)	62.2 (58.1–66.6)	0.1 (-18.2–18.5)	
**Histology**				
Adenocarcinoma				*0.84*
1991–1995	58.6 (54.5–63.0)	53.4 (51.8–55.1)	5.2 (0.6–9.7)	
1996–2000	58.3 (54.3–62.7)	58.9 (57.4–60.5)	-0.6 (-5.0–3.9)	
2001–2005	63.5 (59.7–67.6)	61.1 (59.7–62.4)	2.5 (-1.7–6.7)	
2006–2010	67.5 (63.6–71.6)	64.6 (63.4–65.8)	2.9 (-1.3–7.1)	
2011–2015	69.4 (65.7–73.2)	67.9 (66.8–69.0)	1.5 (-2.4–5.4)	
Neuroendocrine tumour				*0.21*
1991–1995	94.6 (89.7–99.7)	69.4 (56.2–85.7)	25.1 (9.7–40.6)	
1996–2000	94.5 (90.3–98.9)	89.8 (79.6–100.0)	4.7 (-6.9–16.3)	
2001–2005	87.2 (80.8–94.1)	NA[3]	NA	
2006–2010	94.6 (89.9–99.5)	67.5 (55.0–83.0)	27.0 (12.3–41.7)	
2011–2015	86.5 (80.1–93.4)	62.8 (55.2–71.6)	23.6 (13.1–34.2)	
Other				*0.20*
1991–1995	36.6 (19.6–68.5)	NA	NA	
1996–2000	NA	15.1 (10.9–20.8)	NA	
2001–2005	NA	NA	NA	
2006–2010	71.9 (57.8–89.4)	15.1 (11.4–20.0)	56.8 (40.5–73.0)	
2011–2015	70.9 (51.3–98.0)	19.0 (15.1–24.0)	51.8 (28.5–75.2)	

Abbreviations: [1] CI, confidence interval

[2] To test for a difference in survival trend between early- and late-onset patients.

[3] Not available: 5-year survival standardized for age and sex is not available because the survival estimate was not available for all combinations of age and sex.

Supplementary Figure 1. Flowchart of patient cohort

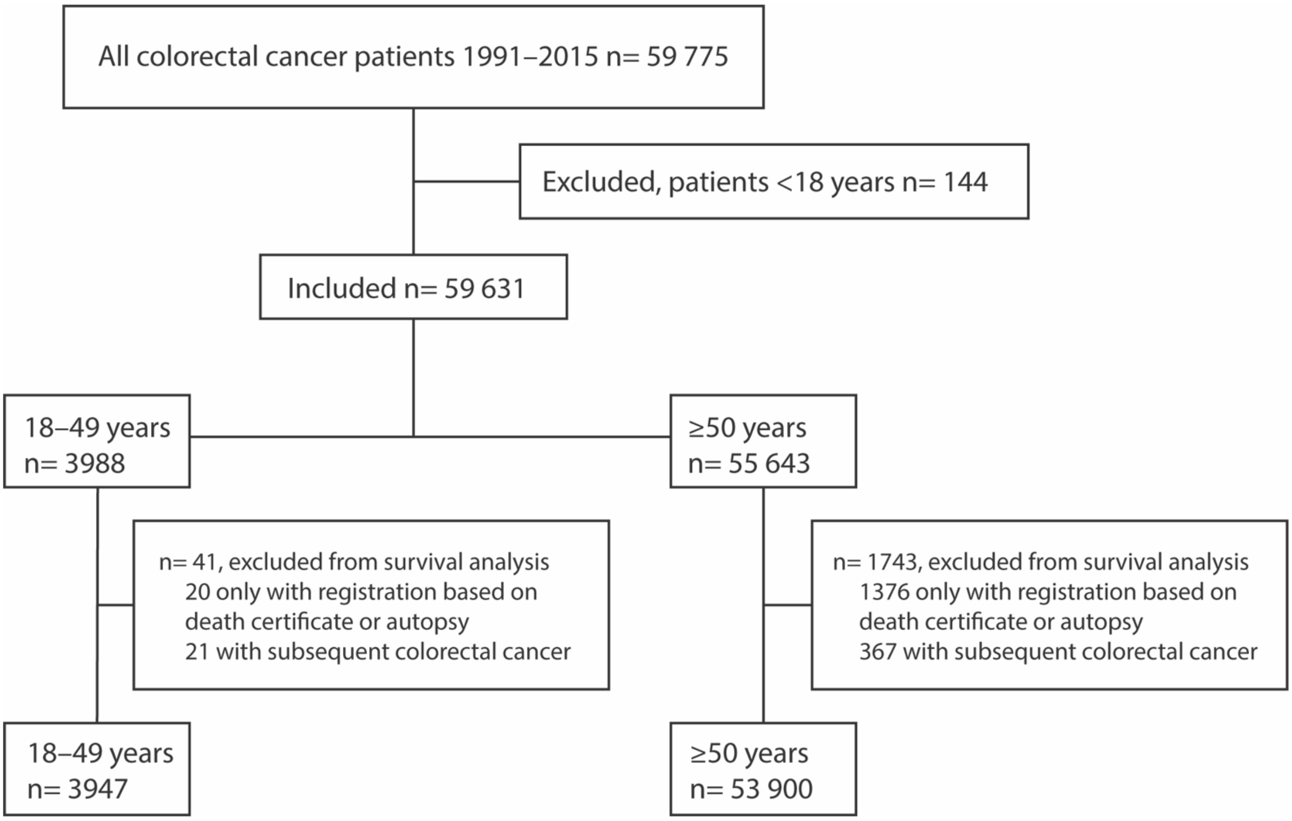

Supplement: Supplementary file 1 [file AO-64-43349-s1.pdf]
